# COMPARATIVE EFFECTIVENESS OF REMOTELY DELIVERED VERSUS IN-PERSON ENHANCE FITNESS AMONG OLDER ADULTS

**DOI:** 10.1093/geroni/igae098.0996

**Published:** 2024-12-31

**Authors:** Kushang Patel, Qian Qiu, Meghan Thompson, Paige Denison

**Affiliations:** University of Washington, Seattle, Washington, United States; University of Washington, Seattle, Washington, United States; Sound Generations, Seattle, Washington, United States; Sound Generations, Seattle, Washington, United States

## Abstract

Access to evidence-based exercise programs, such as Enhance Fitness (EF), is limited for older adults who are homebound or live in rural areas. During the COVID-19 pandemic, EF was adapted for remote delivery (Tele-EF) and implemented nationally; however, its effectiveness is not well established. Therefore, we compared the effectiveness of Tele-EF with standard in-person EF among older adults who had no prior experience with EF. Between 2020-2024, a total of 3,924 older adults had enrollment, attendance, and functional outcome data available in EF’s national database (median time between assessments was 112 days [IQR: 103-126]). Baseline median age was 73 years (IQR: 68-79) and 3,040 (77.4%) were women. The monthly attendance rate in Tele-EF (n=132; mean=66.3% [95% CI: 63.5-69.1%]) and in-person EF (n=3,792; 64.8% [95% CI: 64.2-65.4%]) did not differ significantly (mean difference= 1.5% [95% CI: -2.4%, 5.3%]). The 30-second sit-to-stand test improved significantly from baseline for both Tele-EF (mean difference=3.0 chair stands [95% CI: 2.2-3.8]) and in-person EF (mean difference=1.9 chair stands [95% CI: 1.7-2.1]). Compared with in-person EF, participants in Tele-EF had greater improvement in chair rising performance (p=0.016). For the 8-foot Timed Up & Go (TUG) test, performance improved from baseline for both Tele-EF (mean difference=-0.6 seconds [95% CI: -1.0, -0.3]) and in-person EF (mean difference=-1.3 seconds [95% CI: -1.4, -1.1]). However, improvement in TUG performance did not differ between Tele-EF versus in-person EF (p=0.18). Overall, Tele-EF had similar rates of attendance and effectiveness for improving mobility function in older adults as in-person EF.

